# A Step Forward

**Published:** 1909-11-13

**Authors:** 


### A STEP FORWARD.

We are glad to be able to announce that Lord Braye has at last introduced the Indecent Advertisements (Amendment) Bill into the House of Lords. This Bill has for its object the broadening and amplifying of the salutary clauses in the old Act of 1889. It is sincerely to be hoped that the Lords will find time, even with the imminent distractions of the Finance Bill, to deal generously with this Bill, which is worth cordial support from every thoughtful member of the community, whether lay or professional. The Bill, for example, substitutes for the somewhat loose fifth section of the old Act the following desirable emendation:—"Any advertisement, relating to any disease affecting the generative organs of either sex, or to any complaint or infirmity arising from or relating to sexual intercourse, or to the treatment of any complaint or condition peculiar to females, or to the removal of irregularities in menstruation, and any advertisement relating to drugs, medicines, appliances, or treatment for procuring abortion or promoting miscarriage or preventing conception, or which might reasonably be construed as relating to any illegal medical treatment or to any illegal surgical operation." This is a highly necessary alteration, for, as the law stands, at present such advertisements are allowed to be published and the onus of showing that they are indecent or irregular lies upon the unfortunate moralist (using that term in the sense that Latimer did) who has the courage to bring an action against the paper that has inserted such notices. No such action, as far as we are aware, has ever been brought, probably because the odds are greatly in favour of the defendants in such a case. The Amended Act will improve matters in this direction, solidifying and broadening, as it does, the old fifth clause which has really nothing in its favour. Further on the Bill has the following equally desirable provisions:—2. Whosoever shall exhibit to public view in any house or shop any appliance for procuring abortion or promoting miscarriage or preventing conception, shall, on conviction in manner provided by the Summary Jurisdiction Acts, be liable to a penalty not exceeding five pounds, or, at the discretion of the court, to imprisonment with or without hard labour for a term not exceeding three months. 3. (1) Whosoever shall—(*a*) publish in any newspaper any indecent advertisement; or (*b*) deliver to any other person any indecent advertisement for the purpose of procuring the publication thereof in any newspaper, shall be liable, on conviction in manner provided by the Summary Jurisdiction Acts, to a penalty not exceeding five pounds, or, in the discretion of the court, to imprisonment with or without hard labour for a term not exceeding three months: Provided that on the conviction of a body corporate of an offence against this section the court may impose a penalty not exceeding twenty-five pounds. The value of such restrictions, which are a step in the right direction, can hardly be overestimated when a glance at a Sunday newspaper is sufficient to show the medical reader the amount of indecent rubbish which is foisted upon a gullible public. A certain amount of latitude in the matter of advertisement must be allowed, but of late years the liberty to use the privileges of advertising has degenerated into license, in so far, at least, as notices of matters which are on the borderland between decency and indecency are concerned.

